# Modified per-oral plication of the neo-esophagus for refractory delayed gastric conduit emptying: a novel endoscopic approach

**DOI:** 10.1055/a-2587-9082

**Published:** 2025-05-06

**Authors:** Hugo Uchima, Raquel Muñoz-González, Sofia DallʼOglio, Luis Wong, Harold Benites-Goñi, Ingrid Marin, Elisenda Garsot

**Affiliations:** 1 16514 Hospital Universitari Germans Trias i Pujol, Badalona, Spain; 2 Germans Trias I Pujol Research Institute (IGTP), Barcelona, Spain; 3 16711 Centro Médico Teknon, Barcelona, Spain; 4 33225 Vicerrectorado de Investigación, San Ignacio de Loyola University, Lima, Peru

Delayed gastric conduit emptying (DGCE) affects 15–39% of patients after esophagectomy, increasing the risk of aspiration, pneumonia, and malnutrition 1 . “Sump” formation with chronic dilation often necessitates surgical intervention. Per-Oral Plication of the neo-Esophagus (POPE) is a novel endoscopic technique adapted from endoscopic sleeve gastroplasty (ESG) to remodel the neo-esophagus (and end-stage achalasia megaesophagus) and improve emptying 2 , with 82.3% symptom improvement, although 23.5% requiring repeat intervention.

Studies of ESG have shown that 83.64% of sutures persist at 12 months, with 70.9% maintaining adequate tension 3 . Combining mucosal denudation techniques like argon plasma coagulation (APC) or endoscopic mucosal resection (EMR) may enhance submucosa to submucosa apposition leading to better suture retention and durability 4 5 .

We present a modified POPE by combining mucosal denudation techniques, to address sump-related retention and improve gastric emptying in a patient with refractory DGCE ( Video 1 ).

Modified Per-Oral Plication of the (Neo)Esophagus (POPE) for refractory delayed gastric conduit emptying.Video 1

A 72-year-old woman with esophageal adenocarcinoma underwent partial esophagectomy with gastric conduit reconstruction in 2019. She developed early satiety, worsening heartburn when supine, and progressive oral intolerance, necessitating total parenteral nutrition. Imaging confirmed a neo-esophageal sump with significant retention. Dietary adjustments, prokinetics, and pyloric botulinum toxin injection failed, with worsening symptoms and bile reflux. A multidisciplinary team recommended POPE for sump remodeling.

Modified POPE was performed under general anesthesia in the supine position, using a suturing device without an overtube, combining with EMR and APC. Endoscopic and radiologic assessment confirmed improved conduit alignment and pyloric passage ( Fig. 1 ). The patient was discharged after 48 hours without complications and tolerating diet. At a 4-month follow-up, endoscopy confirmed sustained remodeling with symptom improvement and no retention. In conclusion, modified POPE is a feasible, minimally invasive technique for refractory DGCE due to sump formation. Further studies are needed to evaluate long-term efficacy.

**Fig. 1:**
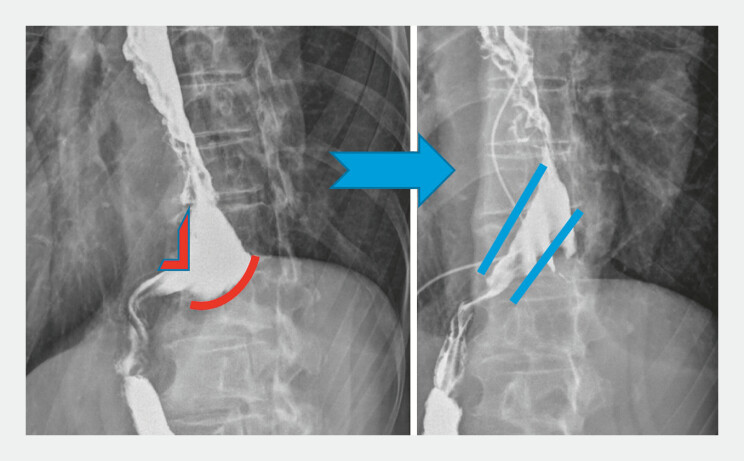
Esophagogram previous to the intervention on the left; esophagogram after POPE on the right. Abbreviation: POPE, per-oral plication of the (neo)esophagus.

Endoscopy_UCTN_Code_TTT_1AO_2AP

## Notes

Endoscopy E-Videos https://eref.thieme.de/e-videos
*E-Videos* is an open access online section of the journal *Endoscopy* , reporting on interesting cases and new techniques in gastroenterological endoscopy. All papers include a high-quality video and are published with a Creative Commons CC-BY license. Endoscopy E-Videos qualify for HINARI discounts and waivers and eligibility is automatically checked during the submission process. We grant 100% waivers to articles whose corresponding authors are based in Group A countries and 50% waivers to those who are based in Group B countries as classified by Research4Life (see: https://www.research4life.org/access/eligibility/ ). This section has its own submission website at https://mc.manuscriptcentral.com/e-videos .
